# The emergence of chromosomally located *bla*_CTX-M_ subtypes in *Salmonella enterica* serotype Kentucky ST198 isolated from diarrhea patients, food, and environmental sources in Henan, China

**DOI:** 10.3389/fmicb.2026.1758643

**Published:** 2026-02-04

**Authors:** Haoyu Qi, Menghan Li, Yanfen Li, Ruichao Li, Ying Cui, Lingling Wu, Meng Zhang, Guangwei Zhang, Yongli Li

**Affiliations:** 1 Henan Center for Disease Control and Prevention, Zhengzhou, Henan, China; 2 Key Laboratory of Food Safety Risk Assessment, National Health Commission of the People’s Republic of China, China National Center for Food Safety Risk Assessment, Beijing, China; 3 Jiangsu Co-Innovation Center for Prevention and Control of Important Animal Infectious Diseases and Zoonoses, College of Veterinary Medicine, Yangzhou University, Yangzhou, China

**Keywords:** *bla*_CTX-M_, multi-drug resistant region, phylogenetic analysis, *Salmonella enterica* serovar Kentucky, ST198

## Abstract

**Introduction:**

Recently, with the resistance to medically important antimicrobial agents, *Salmonella enterica* serovar Kentucky ST198 has attracted continuous attention. In this study, we present the prevalence, antimicrobial resistance (AMR) mechanisms, and comparative genomics study of 68 *Salmonella* Kentucky ST198 isolates exhibiting distinct AMR patterns, from patients, food, and environmental sources in Henan Province, China.

**Methods:**

We evaluated the genomic and antimicrobial resistance characteristics of *Salmonella* Kentucky ST198 obtained from foodborne disease and food safety surveillance in Henan, China, during 2018–2022, using whole-genome sequencing and antibiotic susceptibility testing.

**Results and discussion:**

Among 1,574 *Salmonella* isolates, 68 *S.* Kentucky ST198 isolates were identified, all of which exhibited multi-drug resistance (MDR). Each strain carried between 5 and 19 antimicrobial resistance genes. Phylogenetic analysis revealed that isolates identified in China were clustered into two clades (ST198-1 and ST198-2), characterized by a specific point mutation in the *gyrA* gene and closely related to European isolates. Comparative genomics showed that acquisition of MDR region in clade ST198-2 conferred resistance to azithromycin, fosfomycin, cefotaxime, rifamycin, chloramphenicol, florfenicol, trimethoprim, and ampicillin. Most *bla*_CTX-M_ genes were chromosomally located except one strain carrying *bla*_CTX-M-27_ on plasmid p0111. In clade ST198-2, *bla*_CTX-M-55_ or *bla*_CTX-M-64_ genes were detected within the MDR region, while in clade ST198-1, *bla*_CTX-M-14b_ was inserted into the coding gene clusters of the T6SS, located chromosomally. Considering the extensively drug-resistant nature of the isolates, continuous surveillance and effective measures to control the transmission of *Salmonella* Kentucky ST198 are urgently needed.

## 1 Introduction

Non-typhoidal *Salmonella enterica* (NTS) ranks among the most widespread zoonotic pathogens, with more than 2,600 recognized serovars. Among them, the global spread of multi-drug resistant (MDR) *S.* Kentucky has been a major focus due to its serious threat to livestock and human health, particularly through resistance to medically important antimicrobials, such as fluoroquinolone, extended-spectrum cephalosporins (ESCs), and/or carbapenems (Haley et al., 2019). Furthermore, *S.* Kentucky is strongly associated with the poultry production chain, suggesting a direct link with human infections (Wang et al., 2023).

Initially, *S.* Kentucky was vulnerable to all antibiotics (Hawkey et al., 2019). Since the 1990s, *Salmonella* isolates have showed resistance to ampicillin, streptomycin, gentamicin, sulfamethoxazole, and tetracycline, mediated by the *Salmonella* Genomic Island 1 variant K (SGI1-K) (Weill et al., 2006). Ciprofloxacin resistant (CIP^R^) *S.* Kentucky was subsequently reported in North Africa (Le Hello et al., 2011). Resistance evolved rapidly, shifting from absence of resistance before 1990 to a sharp increase in CIP^R^ isolates at the beginning of the 21st century, rising from 55% in 2007 to 88% in 2017 (European Food Safety Authority and European Centre for Disease Prevention and Control, 2020; Le Hello et al., 2013). CIP^R^ isolates emerged in *S.* Kentucky due to specific point mutations in quinolone-resistance determining regions (QRDRs) of the *gyrA* and *parC* genes and/or acquisition of plasmid-mediated quinolone resistance (PMQR) genes, including *aac(6′)lb-cr, oqxAB, qepA, qnrA, qnrB, qnrC, qnrD, and qnrS* (Hooper and Jacoby, 2016; Cuypers et al., 2018). Multilocus sequence typing (MLST) showed that CIP^R^ *S.* Kentucky isolates belonged to sequence type 198 (ST198) (Biggel et al., 2022), the most prevalent sequence type associated with human infections (Vázquez et al., 2021; Chen et al., 2021). In recent years, the extend-spectrum β-lactamase (CTX-M and TEM), cephalosporinase (CMY), or carbapenemase (OXA-48, VIM, and NDM) encoding genes have been detected in CIP^R^ *S.* Kentucky ST198 (Le Hello et al., 2013; Hawkey et al., 2019; Alghoribi et al., 2020). Ciprofloxacin and extended-spectrum cephalosporins (ESCs) remain the most common antibiotics used for treating non-typhoidal *Salmonella* (NTS) infections in humans. Further, carbapenemase represent the last-line option for treating serious infection of ESCs-resistant Gram-negative bacteria. Thus, resistance to ciprofloxacin, cephalosporins or carbapenemase in *S.* Kentucky ST198 constitutes a serious public health concern, potentially leading to treatment failures.

Although, *S.* Kentucky ST198 has been sporadically reported in China, understanding its genomic landscape and antimicrobial resistance (AMR) profiles is crucial in preventing transmission of these MDR pathogens. Here, we present the genomic epidemiology and AMR data of 68 *S.* Kentucky ST198 isolates collected from foodborne disease and food safety surveillance in Henan, China, between 2018 and 2022. Using antimicrobial susceptibility testing and whole-genome sequence (WGS) analysis, we determined the prevalence and mechanisms of resistance and conducted a genomic comparative genomic analyzes to identify potential drivers of variation in AMR profiles and clonality.

## 2 Materials and methods

### 2.1 Bacteria isolates and serovar detection

A total of 68 *S.* Kentucky ST198 isolates were identified from 1,574 cultured *Salmonella* isolates from diarrhea patients, food, and environmental samples across eleven regional Center for Disease Control and Prevention diagnostic laboratories located in Henan, China, between 2018 and 2022. Serovars of confirmed *Salmonella* isolates were determined by slide agglutination using commercial antisera (Statens Serum Institute, Denmark), following the Kauffmann–White scheme.

### 2.2 Antimicrobial susceptibility testing

Antimicrobial susceptibility testing of 68 *Salmonella* Kentucky ST198 isolates was performed against 15 agents from 11 distinct antimicrobial classes using the agar dilution method (Clinical Laboratory Standards Institute (CLSI), 2022), with *Escherichia coli* ATCC™ 25922 as the quality control strain. The agents and classes include: penicillin (ampicillin, AMP); β-lactam/β-lactamase inhibitor combinations (ampicillin-sulbactam, AMS); cephalosporins (cefotaxime, CTX; ceftazidime, CAZ; cefazolin, CFZ; and cefoxitin, CFX); folate pathway inhibitors (trimethoprim-sulfamethoxazole, SXT); carbapenems (imipenem, IPM); aminoglycosides (gentamicin, GEN); tetracyclines (tetracycline, TET); macrolides (azithromycin, AZM); polymyxins (colistin, CT); phenicols (chloramphenicol, CHL); and quinolones (ciprofloxacin, CIP; and nalidixic acid, NAL). MDR phenotype was defined as resistance to three or more of these antimicrobial classes (Magiorakos et al., 2012).

### 2.3 Whole-genome sequencing, *de novo* assembly, and annotation

Genomic DNA from *Salmonella* was extracted using the QIAamp DNA Mini Kit (QIAGEN, Hilden, Germany). All DNA samples were sequenced on the Illumina NovaSeq 6,000 platform (Illumina, USA), generating 150-bp paired-end reads from libraries with an average insert size of 350-bp and sequencing depth of more than 100×. Raw reads were filtered to remove low-quality sequences and subsequently assembled *de novo* using SPAdes v3.8.2 (Bankevich et al., 2012). Five representative isolates were further sequenced by long-read sequencing using the MinION platform from Oxford Technologies (ONT, Oxford, UK). Complete genome sequences were obtained through a hybrid *de novo* assembly strategy with Unicycler (Wick et al., 2017). Draft and complete genomes were annotated using Prokka (Seemann, 2014). AMR genes and chromosomal point mutations were identified using ResFinder4.1 with thresholds of 90% identity and ≥80% minimum length coverage (Bortolaia et al., 2020). The replicon types of plasmids and insertion sequences were detected using PlasmidFinder database and ISFinder database under the same thresholds (Carattoli et al., 2014; Kichenaradja et al., 2010). Core genome multilocus sequence typing (cgMLST) profiles (cgSTs) of the 68 isolates were analyzed based on 3,002 alleles using the cgMLSTFinder database (Alikhan et al., 2018).

### 2.4 Phylogenetic analysis based on core-genome single nucleotide polymorphisms (SNPs)

Core genomes of all assemblies were calculated using Roary v3.13.0 (Page et al., 2015). Snippy v4.6^1^ were used to clean indels in the core genomes, while Gubbins v3.3.1 (Croucher et al., 2015) were used to exclude recombination region. Core genome single nucleotide polymorphisms (cgSNPs) were extracted using SNP-sites v2.5.1 (Page et al., 2016). SNP distance matrices for all isolates were generated using snp-dist v.0.8.2.^2^ Non-repetitive core SNPs were used to construct phylogenetic analysis by the maximum likelihood method with FastTree v2.1.11 (Price et al., 2009) and visualized using ChiPlot (Xie et al., 2023). In addition, 168 published genomes of *S*. Kentucky ST198 isolates from other regions were downloaded from the National Center for Biotechnology Information (NCBI) and incorporated into the phylogenetic tree with *S*. Kentucky ST198 isolates of Henan using cgSNPs.

## 3 Results

### 3.1 Sources of Chinese *S.* Kentucky ST198 isolates and their antimicrobial susceptibility profiles

From 2018 to 2022, a total of 1,574 *Salmonella* isolates were collected from diarrhea patients, food, and environmental samples from 11 cities in Henan, through the laboratory-based foodborne disease and food safety surveillance system in China. Among them, 102 serovars were detected (Supplementary Table S1), with *S*. Kentucky ranking the fourth most common serovar (Table 1).

**Table 1:** The serovars of *Salmonella* strains isolated from different sources.

Serovars	Sources (no.)			Total
	Diarrhea patients	Food	Environment	
*S*. Enteritidis	416	127	10	553
*S*. Typhimurium	269	47	–	296
*S.* Newport	3	63	13	79
*S.* Kentucky	19	48	9	76
*S*. Corvallis	4	34	11	49
*S.* Thompson	37	9	1	47
*S.* Agona	9	32	1	42
*S.* Schwarzengrund	6	16	4	26
*S*. Indiana	4	19	–	23
*S.* Derby	15	7	–	22
*S.* London	18	4	–	22
*S.* Mbandaka	6	2	12	20
*S.* Glodcoast	17	1	–	18
*S.* Stanley	14	4	–	18
*S.* Infanti	7	7	–	14
*S.* Rissen	6	6	–	12
*S.* Typhi	9	1	–	10
*S.* Muenster	2	5	2	9
*S.* Braenderup	1	8	–	9
*S.* Cyprus	–	8	–	8
*S.* Senftenberg	3	5	–	8
Others	80	95	18	193
Total	945	548	81	1,574

Of these *S. enterica* strains, a total of 76 *S.* Kentucky isolates (4.8%, 76/1574) were identified, including 68 ST198 (89.5%, 68/76) and 8 ST314 (10.5%, 8/76) isolates. The ST198 strains were isolated from diverse sources, including patients (*n* = 18), poultry products (*n* = 38), beef (*n* = 1), pork (*n =* 1), and environment (*n =* 10) (Supplementary Table S2). The poultry products isolates (*n =* 38) were obtained from broiler chicken in retail market (*n =* 25), chicken farm (*n =* 1), and slaughterhouse (*n =* 12). The environmental isolates (*n =* 10) were obtained from chicken farm (*n =* 2) and slaughterhouses (*n =* 8).

Antimicrobial susceptibility testing of the 68 *S.* Kentucky ST198 isolates against a panel of 15 antimicrobial compounds revealed resistance to 14 compounds (Supplementary Table S3). All the isolates were MDR strains, showed resistance to three or more classes of antimicrobials. No resistance phenotype was detected against imipenem. Resistance to nalidixic acid (100%) and ciprofloxacin (100%) was the most common, followed by ampicillin (98.5%), tetracycline (98.5%), cefotaxime (97.1%), cefazolin (97.1%), gentamicin (91.2%), chloramphenicol (83.8%), ceftazidime (82.4%), sulfamethoxazole-trimethoprim (77.9%), and azithromycin (70.6%). Resistance to colistin (2.9%) and cefoxitin (8.8%) was rare.

### 3.2 Phylogenetic relationship and molecular characteristics of *S.* Kentucky ST198 isolates

**Figure 1:**
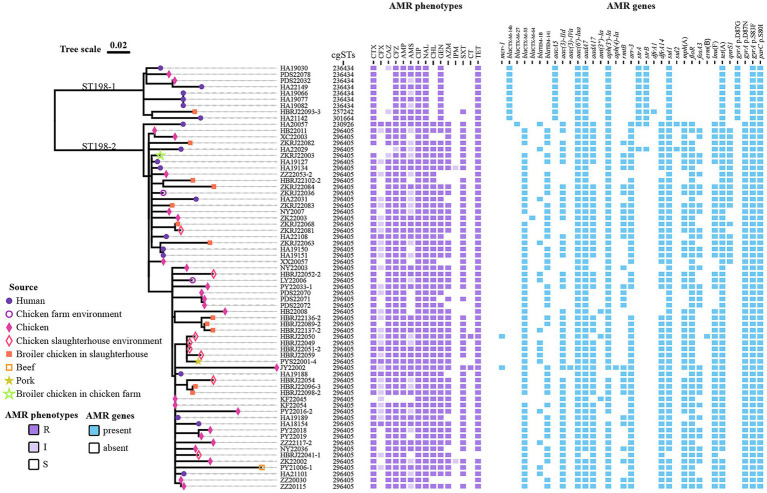
Phylogenetic tree of the 68 *S.* Kentucky ST198 isolates from Henan, China. A maximum likelihood tree is constructed based on single nucleotide polymorphisms (SNPs) in the core genome of 68 *S.* Kentucky ST198 isolates. ST198-1 and ST198-2 are the two clades of ST198. Tips of the tree are colored according to source (see key). The following information is presented to the left of the isolate IDs: the cgSTs, antimicrobial resistance (AMR) phenotype, and presence/absence of AMR gene.

To investigate the genetic relationship of Chinese isolates, we executed a phylogenetic analysis based on cgSNPs. The Chinese isolates grouped into two groups, namely ST198-1 (9 isolates) and ST198-2 (59 isolates) with distinct QRDR mutations in *gyrA* gene (Figure 1). The clade ST198-1 isolates originated from human (*n =* 6), chicken in market (*n =* 2), and slaughterhouse (*n =* 1) (Figure 1, Supplementary Table S2). The isolated sources of clade ST198-2 strains were human (*n =* 12), samples in retail market (chicken, beef and pork, *n =* 25) and samples in farm or slaughterhouses (broiler chicken *n =* 12 and environment *n* = 10). Within subclade ST198-2, pairwise cgSNP differences among strains isolated from chickens and the environmental samples across different stages (incubation, breeding, or slaughtering) within the same slaughterhouse ranged from 0 to 43 (Supplementary Table S7). Furthermore, cgSNP differences between strains isolated from diarrhea patients and poultry products in local supermarkets ranged from 10 to 18. Most strains of clade ST198-2 were resistant to azithromycin (81.4%) and trimethoprim (88.1%), whereas all ST198-1 strains were sensitive, except for one trimethoprim resistant strain (Supplementary Table S2).

The cgMLST was analyzed based on the whole genome sequences to investigate molecular characteristics of *S.* Kentucky ST198 isolates. Among the *S.* Kentucky ST198 isolates were grouped into five cgSTs (Figure 1) and among ST198-1 isolates, three cgSTs were detected, with cgST236434 predominating across four cities (Kaifeng, Pingdingshan, Nanyang, and Zhoukou) of Henan (Supplementary Table S2). Among ST198-2 isolates, two cgSTs detected were [cgST230926 98.3% (58/59)] and [cgST296405 1.7% (1/59)]. The predominant cgST296405 were distributed across 10 cities (Hebi, Xuchang, Zhoukou, Nanyang, Kaifeng, Luoyang, Pingdingshan, Puyang, and Jiyuan) in Henan.

**Figure 2:**
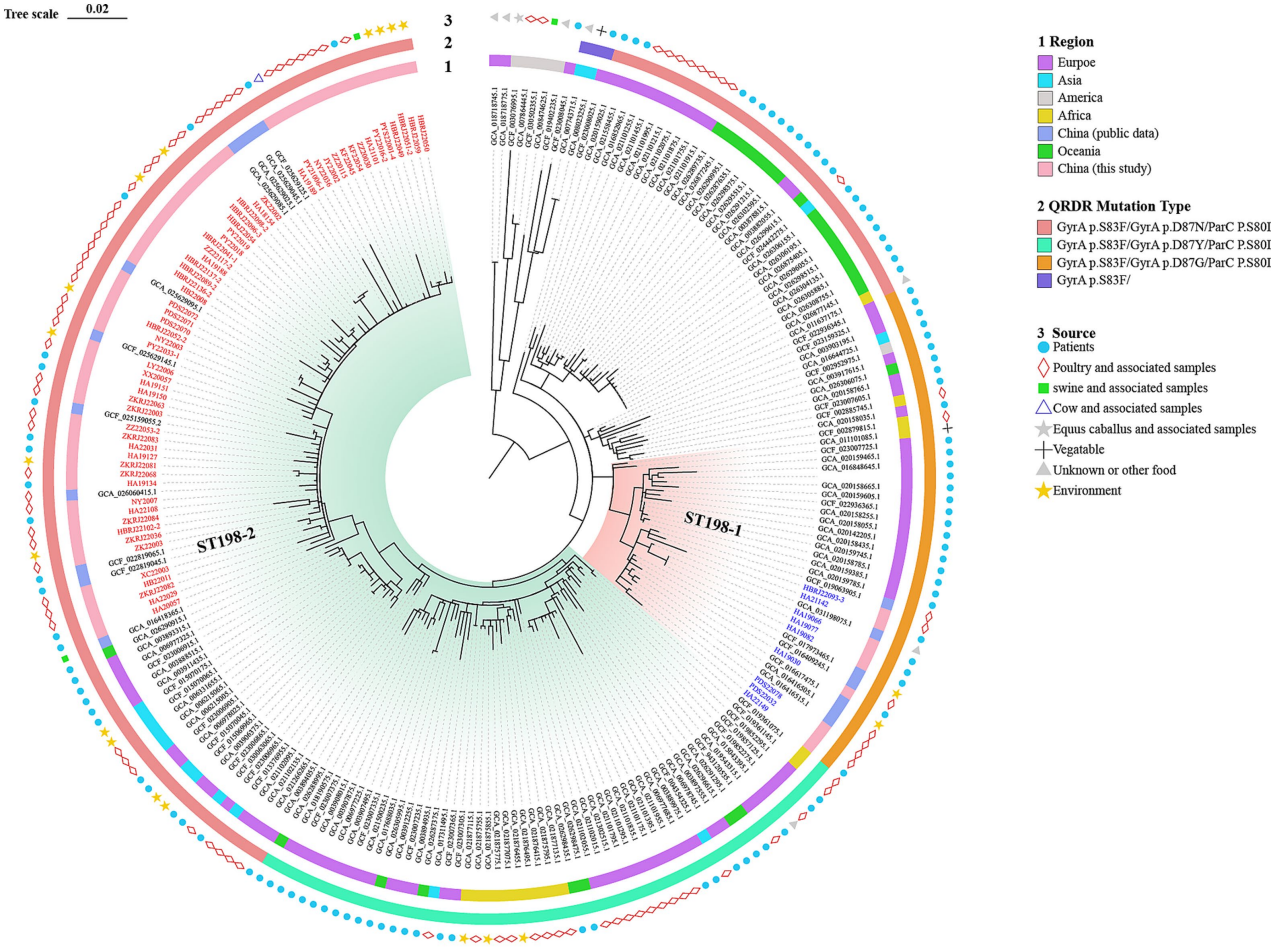
Core genome phylogeny of 236 global *S.* Kentucky ST198 isolates. Geographic origin (ring 1), mutation type in quinolone resistance-determining regions (QRDRs) (ring 2), and source (ring 3) are shown. Clade ST198-1 is shaded in light red and clade ST198-2 is shaded in light green. Branches corresponding to isolated from this study are highlighted in blue (ST198-1) and red (ST198-2).

To investigate the genetic relatedness of ST198 isolates globally, we constructed a phylogenetic tree combining our isolates with 168 ST198 genomes obtained from the NCBI database. Consistent with previous reports, global ST198 isolates were grouped into multiple clusters (Figure 2). The phylogenetic relationship showed that the global ST198 isolates clustered separately with distinct QRDR mutations in *gyrA* gene. Additionally, the clade ST198-1 of Henan clustered with strains from Europe (*n =* 15), other provinces in China (*n =* 7), and Africa (*n =* 1) (Figure 2, Supplementary Table S4). The Henan ST198-2 clade clustered with strains from Europe (*n =* 47), Africa (*n =* 12), Asia (*n =* 11), other provinces in China (*n =* 11), and Oceania (*n =* 8). Overall, Chinese isolates were mostly related to the European isolates, with ST198-2 isolates showing stronger genetic relationship with strains from Europe than ST198-1.

### 3.3 Prevalence and differences of resistance genes and mutations among Chinese *S.* Kentucky isolates

Among the 68 isolates, 31 acquired AMR genes were detected, along with a single-point mutation in *gyrA* and *parC* (Figure 1). All isolates harbored multiple aminoglycoside resistance genes. Four *bla*_CTX-M_ subtypes were identified among the 66 ESBL-positive *Salmonella* isolates, including *bla*_CTX-M-14b_ (*n* = 9), *bla*_CTX-M-55_ (*n* = 55), *bla*_CTX-M-64_ (*n* = 1), and *bla*_CTX-M-27_ (*n* = 1). The *bla*_TEM_ subtypes encoding resistance to ampicillin were also detected among the ST198 isolates, including *bla*_TEM-1B_ (*n* = 15) and *bla*_TEM-141_ (*n* = 40). For fluoroquinolone resistance, all the isolates carried multiple single-point mutations in *gyrA* and *parC* (GyrA S83F, ParC T57S, and ParC S80I). Additionally, different single-point mutations were detected in *gyrA* including D87N (*n* = 59) and D87G (*n* = 9) along with the plasmid-mediated quinolone resistance (PMQR) gene, including *qnrS1* (*n* = 19). Alleles of dihydrofolate reductase (*dfr*)-encoding genes that endowed resistance to trimethoprim were noticed in 54 isolates, comprising two different variants, *dfrA14* (*n* = 53) and *dfrA1* (*n* = 1). In addition, six types of acquired AMR genes were also present in the majority of the isolates. These included *tet*(A) (*n* = 67, encoding resistance to tetracycline), *sul1* (*n* = 67, sulphonamides), *floR* (*n* = 58, phenicols), *arr-3* (*n* = 53, rifamycin), *Inu*(F) (*n* = 51, lincomycin), and *mph*(A) (*n* = 48, azithromycin). Streptomycin resistant genes *strA* and *strB* were detected in 11 ST198 isolates. Importantly, the emerging mobile colistin resistance gene *mcr-1* was also detected in two isolates.

The distribution of resistance genes clearly differed between ST198-1 and ST198-2 isolates (Figure 1). Acquired AMR genes conferring resistance to fluoroquinolone (*qnrS1*), third/fourth generation cephalosporins (*bla*_CTX-M-55_, *bla*_CTX-M-64_, and *bla*_CTX-M-27_), ampicillin (*bla*_TEM-1B_, and *bla*_TEM-141_), aminoglycosides (*aac(3)-IId*, *aac(3)-IVa*, *aadA17*, *ant(3″)-Ia*, *aph(4)-Ia*, and *rmtB*), rifamycin (*arr-3*), trimethoprim (*dfrA14*), sulphonamides (*sul2*), lincomycin (*ermB* and *Inu*(F)), phenicols (*floR*), fosfomycin (*fosA3*), and colistin (*mcr-1*) were only detected in ST198-2 isolates. In contrast, resistance genes for third/fourth generation cephalosporins (*bla*_CTX-M-14b_), aminoglycosides (*aacA5*), and trimethoprim (*dfrA1*) were restricted to ST198-1 isolates (Supplementary Table S6). Genes *strA* and *strB* encoding streptomycin resistance were more predominant in ST198-1. For multiple single-point mutations detected in *gyrA*, different amino acid substitutions were more common in each clade; GyrA D87G was predominant in ST198-1, while GyrA D87N was predominant in ST198-2.

### 3.4 Location and genetic structure of *bla*_CTX-M_ genes among Chinese *S.* Kentucky isolates

**Figure 3:**
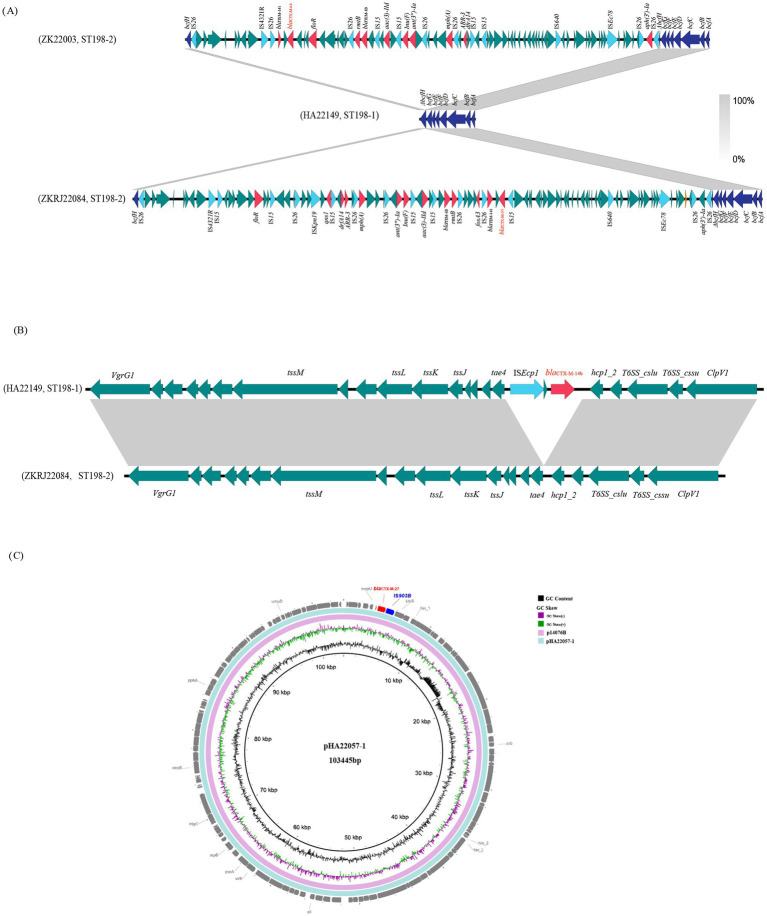
Location and genomic structure of *bla*_CTX-M_ genes in *S.* Kentucky ST198 isolates. **(A)** Linear genetic structure comparison of the MDR region inserted into *bcfH* gene in representative ST198-2 isolates. The matched regions between two sequences are shown in light gray blocks. The arrows indicate genes related to resistance and transfer (red: resistance genes; light blue: insertion sequences (IS); dark blue: *bcfABCDEFGH* gene clusters; green: other functions). **(B)** Linear genetic structure comparison between chromosome of representative ST198-1 isolates harboring the chromosomal *bla*_CTX-M-14b_ and ST198-2 isolates which are non-*bla*_CTX-M-14b._ The matched regions between two sequences are shown in light gray blocks. The arrows indicate genes related to resistance and transfer [red: resistance genes; light blue: insertion sequences (IS); green: other functions]. **(C)** Comparison between p0111 plasmids harboring *bla*_CTX-M-27_ identified in this study (pHA22057-1) with reference plasmid (p14076B) from online NCBI database.

The distribution of *bla*_CTX-M_ subtypes genes differed between isolates in ST198-1 and ST198-2 (Figure 1). The ST198-1 strains carried *bla*_CTX-M-14b_, while ST198-2 strains carried *bla*_CTX-M-55_, *bla*_CTX-M-27_, and *bla*_CTX-M-64_. To further confirm the genetic context of resistance genes, we determined the complete genomes of five representative Chinese isolates from the two clades by long-read sequencing and obtained complete and circular genome sequences. The architecture of the antibiotic resistance complement was complex, with multiple locations in the bacterial genome harboring antibiotic resistance genes. Further analysis showed that *bla*_CTX-M-14b_, *bla*_CTX-M-55_, and *bla*_CTX-M-64_ genes were chromosomally located on different regions in the chromosome whereas *bla*_CTX-M-27_ bounded by IS*903B* was detected in a p0111 plasmid (denoted as pHA22057-1) (Figure 3). In ST198-1 isolates, genomic alignment showed that *bla*_CTX-M-14b_ was located on a 2848-bp translocatable unit mediated by IS*ECP1*, inserted into the coding gene clusters of the type VI secretion system (T6SS) in the chromosome (Figure 3A). The 2848-bp unit was absent in the chromosome among ST198-2 isolates (non-*bla*_CTX-M-14b_). Among ST198-2 isolates, both *bla*_CTX-M-55_ and *bla*_CTX-M-64_ genes were detected in a MDR region (Figure 3B). The MDR region was inserted into *bcfH* gene at downstream of *bcfABCDEFG*, disrupting *bcfH*. This MDR region, absent in the chromosome among ST198-1 isolates, carried multiple resistance genes (*qnrS1*, *bla*_TEM-1B_, *bla*_TEM-141_, *aac(3)-IId*, *rmtB*, *arr-3*, *dfrA14*, *Inu*(F), *floR*, *and fosA3*) detected in ST198-2 isolates. The molecular structures and resistance genes of MDR regions were different due to the plasticity of the MDR region whereas the sequence comparison indicated similar backbone structures. The MDR chromosomal regions were bilaterally flanked by IS*26* containing multiple insertion sequences that may contribute to the spread of antibiotic resistance genes. These MDR regions harbored 10–14 resistance genes, including *bla*_CTX-M-55_/*bla*_CTX-M-64_, *bla*_TEM-141_, *bla*_TEM-1B_, *aph(3′)-Ia*, *ant(3″)-Ia*, *aac(3)-IId*, *fosA*, *rmtB*, *lnu*(F), *mph*(A), *dfrA14*, *arr-3*, *qnrS1*, and *floR.* In this study, *bla*_CTX-M-27_ was the only plasmid-borne *bla*_CTX-M_ subtype identified among the ST198 isolates from China. This gene, mediated by IS*903B*, was carried on a 103,445-bp plasmid belonging to the p0111 type (designated pHA22057-1) (Figure 3C).

### 3.5 Structure of SGI1-K among Chinese *S.* Kentucky isolates

**Figure 4:**
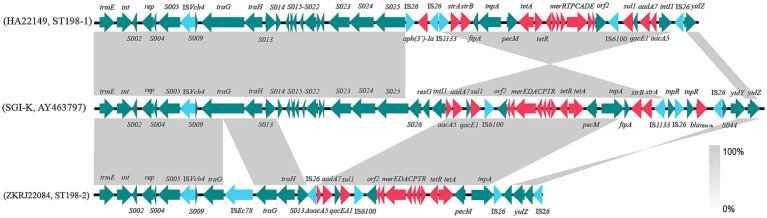
Genetic structure of SGI1-K variants of *S.* Kentucky ST198 isolates. Matched regions between two sequences are shown in light gray blocks. The arrows indicate genes related to resistance and transfer [red: resistance genes; light blue: insertion sequences (IS); green: other functions].

Two slightly different SGI1-K variants (type I and II) were detected in *S.* Kentucky strains in this study (Figure 4). Additionally, type I and type II were detected in clades ST198-1 and ST198-2, respectively. The variant type I of SGI1-K contains several deletions compared to the prototype. The deleted regions include *S026*, *resG*, the *tnpR* gene of Tn5393, a *bla*_TEM-1b_-bearing Tn2, and *ΔS044*. One IS*26* composite-like transposonal module were inserted in downstream of *S025* gene, consisting of IS*26*-[*aph(3′)-Ia*]-IS*26*. Compared to the prototype SGI1-K, IS*Ec78* was inserted into the backbone gene *traG*, and a large absence of backbone genes were detected in the SGI1-K variant type II. The MDR region of SGI1-K variants type II was bounded by two copies of IS*26* with opposite orientation. Regarding the repertoire of antimicrobial resistance genes, the variant type I maintained *strAB*, *tet*(A), *sul1*, *aadA7*, and *aacCA5*, whereas the variant type II carried only *tet*(A), *sul1*, and *aadA7*.

### 3.6 Plasmids in Chinese *S.* Kentucky isolates

Plasmid replicons belonging to 15 different plasmid types were detected in 68 Chinese *S.* Kentucky isolates. ColRNAI-type (*n =* 63) plasmid was the most predominant one, followed by Col156 (*n =* 44) and Col440I (*n =* 39). Eight different plasmid incompatibility types (IncI1, IncFIB, IncX1, IncR, IncY, and IncI2) were also identified among 29 isolates. In addition to the a 103445-bp p0111 plasmid (Figure 3C) harboring *bla*_CTX-M-27_ gene mentioned above, three plasmid incompatibility types (IncI1, IncY, and IncI2) carrying additional AMR genes were identified among the complete genomes of five representative Chinese isolates. One 103079-bp IncI1 plasmid harbored *ermB* gene conferring resistance to lincomycin. One 97831-bp IncY plasmid was found to carry a 4,748-bp transposon Tn6330(IS*Apl1*-*mcr-1*-*pap2*-IS*Apl1*), whereas another *mcr-1* gene was found in a 60793-bp IncI2 plasmid.

## 4 Discussion

Prevalence of *S.* Kentucky ST198 isolates has been reported as a global public health concern, especially in Europe, East Africa, and the United States (European Food Safety Authority and European Centre for Disease Prevention and Control, 2020; Saraiva et al., 2022; Tate et al., 2022). *S.* Kentucky ST198 appears to adapt readily to antibiotic selection pressures in various environments, steadily accumulating genetic elements that confer resistance to multiple antibiotic classes, including the last-line clinical agents (Le Hello et al., 2011, 2013; Hawkey et al., 2019). Accordingly, *S.* Kentucky ST198 should be considered as a high-risk global MDR clone associated with both animal (especially poultry) and human infections. China, a major poultry consumer, has correspondingly reported a high incidence of *S.* Kentucky ST198 in poultry (Gu et al., 2020). Therefore, understanding the genomic epidemiology and transmission dynamics of *S.* Kentucky ST198 at both local and global scales is of great importance. The findings of this study demonstrate the genomic and resistance characteristics of *S.* Kentucky ST198 and highlight the emergence of chromosomally located *bla*_CTX-M_ subtypes genes along with various resistance genes.

Previous studies have identified slaughter as a major contributor to contamination in retail meat (Xiong et al., 2020; Caffrey et al., 2021). Genetic profiles of ST198 isolates from the farm, slaughterhouse, and supermarket were highly comparable, all tracing back to the local broiler production network. These findings highlight the poultry supply chain as a plausible route for the clonal spread and cross-contamination of this lineage, warranting further investigation. Human infections are likely acquired by the consumption of contaminated poultry, reinforcing poultry as the main risk food for ST198 transmission (She et al., 2023; Wang et al., 2023).

Phylogenetic analysis revealed two major clades, namely ST198-1 and ST198-2, among Chinese *S.* Kentucky ST198 isolates. The predominant cgMLST type of ST198 strains of Henan Province was cgST296405, distinct from other provinces (cgST230926, Zhejiang, Fujian, Anhui, Guangdong, Liaoning, etc.) (Wang et al., 2023). This indicated that cgST296405 strains of ST198 were locally endemic in Henan. According to the global phylogenetic relatedness of *S.* Kentucky ST198, isolates identified in China were mostly closely related to the European isolates. The global isolates may cluster separately with distinguished QRDR mutations in the *gyrA* gene. Isolates encoding GyrA D87G (Asp87Gly) and D87Y (Asp87Tyr) formed two monophyletic branches. The isolates encoding GyrA D87N emerged in paraphyletic branches embedded within the D87Y cluster. These QRDR mutation-specific branches remain consistent with previous studies (Mahindroo et al., 2019; Biggel et al., 2022).

The distribution of resistance genes was clearly different between isolates in ST198-1 and ST198-2, primarily due to the MDR region inserted into the chromosome of ST198-2 isolates. The MDR region shared similar backbone structures and harbored 10–14 AMR genes (*mph*(A), *bla*_CTX-M-55_/ *bla*_CTX-M-64_, *qnrS1*, *bla*_TEM-1B_, *bla*_TEM-141_, *aac(3)-IId*, *ant(3″)-Ia*, *aph(3′)-Ia*, *rmtB*, *arr-3*, *dfrA14*, *Inu*(F), *floR*, *and fosA3*), conferring resistance to azithromycin, cefotaxime, chloramphenicol, rifamycin, trimethoprim, ampicillin, florfenicol, and fosfomycin. These regions were rich in ISs and transposons, with each MDR region carrying one or more copies of IS*26*. The IS*6* family, a clinically important group of insertion sequences including IS*26* has proved to be instrumental in the rearrangement and spread of multiple antibiotic resistance found in many Enterobacterial isolates as both chromosomal and plasmid components of this family have received particular attention for their clinical impact (Jiang et al., 2020; Varani et al., 2021; Partridge et al., 2018). The MDR region differed by insertions, deletions, and rearrangements of multiple segments involving resistance genes, likely mediated by mobile elements such as IS*26*.

Our findings highlighted the increasing prevalence of MDR *S.* Kentucky in China, along with the differential characteristics of resistance gene acquisition in various lineages. Among ST198-1 isolates, *bla*_CTX-M-14b_ mediated by IS*ECP1* was located on the chromosome, whereas in ST198-2 isolates, *bla*_CTX-M-55_, *bla*_CTX-M-64_, *bla*_TEM-1B_, and *bla*_TEM-141_ genes were detected within the MDR region, which was inserted into the *bcfH* gene on the chromosome, disrupting its function. This study reports the first instance of chromosomal insertion of the *bla*_CTX-M-64_ gene in *S.* Kentucky isolates. Extend-spectrum β-lactamases (ESBLs) represent the major mechanism of resistance to β-lactamase antibiotics in Enterobacteriaceae. CTX-M-type and TEM-type ESBLs genes have been reported as the predominant types of ESBLs, mainly mediated by plasmids, with the exception of *bla*_CTX-M-14b_, which was located on the chromosome (Cao et al., 2021; Coipan et al., 2020). Similarly *bla*_CTX-M-14b_ has been found in clones increasingly associated with human infections in Europe and in isolates from animal sources across Europe, Africa, and Asia (Lei et al., 2020; Coipan et al., 2020). Additionally, chromosomally located *bla*_CTX-M-14_ or *bla*_CTX-M-55_ have been detected in *Salmonella* Indiana, Chester, and Typhimurium among Chinese isolates (Zhang et al., 2019; Du et al., 2022). The transfer of CTX-M-type and TEM-type ESBLs genes from plasmids to chromosomes may contribute to their long-term retention, as chromosomal genes are less prone to loss during bacterial replication compared to plasmid-borne genes. The emergence of chromosomally located ESBLs genes may attributed to strong selective pressure from antibiotic use in poultry, the primary reservoir of *S.* Kentucky (Hawkey et al., 2019). In this study, multiple resistance genes were integrated into the chromosome of *S.* Kentucky ST198-2 strains. If stably integrated, these chromosomal genes could be vertically transferred as intrinsic components of the MDR lineage, facilitating persistence and dissemination of resistance. However, direct evidence for the stability of these chromosomally integrated genes requires further longitudinal tracking or experimental validation. More importantly, these strains can acquire more resistance genes mediated through mobile elements, leading to an extensively drug-resistant *S.* Kentucky strain, which poses a serious public health threat.

Additionally, in this study two variants of SGI1-K were detected, mainly corresponding to the two clades, with extensive deletions observed in clade ST198-2. Previous studies have reported numerous SGI1-K variants of *S.* Kentucky ST198 globally, with diverse structures indicating rapid evolution within this clone, mainly due to insertions, deletions, or rearrangements of backbone genes and resistance modules mediated by IS*26* (Hawkey et al., 2019).

Genomic analysis revealed that different known plasmids (p0111, IncI1, IncY, and IncI2) carried additional resistance genes among Chinese *S.* Kentucky ST198 isolates, conferring resistance to cephalosporins, lincomycin, or colistin. Despite the high frequency of resistance genes, no carbapenem-resistance genes (*bla*_OXA-48_ and *bla*_NDM-1_) previously reported on IncI1 plasmid in *S.* Kentucky ST198 isolates (Hawkey et al., 2019; Alghoribi et al., 2020), were detected in this study. However, IncHI2 plasmids harboring numerous resistance genes in *S.* Kentucky ST198 isolates were reported in Spain and China (Chen et al., 2021; Samper-Cativiela et al., 2022). IncHI2 plasmids are important conjugative vectors for critical resistance genes such as *bla*_CTX-M_, *oqxAB*, *qnrB*, and *mcr*; these are widespread among the members of the Enterobacteriaceae family (Macesic et al., 2021; Li et al., 2014; García-Fernández and Carattoli, 2010; Mu et al., 2022). This raises the concern that the high-risk clone ST198 may become more resistant through dissemination of MDR plasmids, further complicating treatment strategies in China.

## 5 Conclusion

In this study, we report the genomic and resistance characteristics of *S.* Kentucky ST198 in Henan, China. Compared to ST198-1, acquisition of an MDR region render the ST198-2 *S.* Kentucky isolates extensively drug-resistant, requiring close monitoring as a high-risk clone. Numerous AMR genes, especially *bla*_CTX-M-14b_, *bla*_CTX-M-55_, and *bla*_CTX-M-64_, were inserted into the chromosome of *S.* Kentucky through insertion sequences. Mobile elements, plasmids, and insertion sequences play a crucial role in facilitating the dissemination of AMR genes among ST198 isolates. However, the stability of these chromosomally integrated resistance genes remains to be confirmed through additional longitudinal or experimental studies. The severe resistance profile of *S.* Kentucky ST198 may further accumulate last-line resistance determinants, potentially leading to clinical treatment failure. Therefore, continuous genomic investigation and surveillance are essential to extend our studies focusing on the transmission dynamics and evolutionary strategies of these ST198 isolates.

## Data Availability

The complete genome sequences of were submitted to NCBI database under the accession numbers CP174483-CP174493 and CP175711-175719. Whole-genome sequencing rawdata was deposited in the NCBI Sequence Read Archive database (BioProject number: PRJNA1188319).

## Footnotes

1
https://github.com/tseemann/snippy

2
https://github.com/tseemann/snp-dists
