# Supplementary material for: The emergence of chromosomally located blaCTX-M subtypes in Salmonella enterica serotype Kentucky ST198 isolated from diarrhea patients, food, and environmental sources in Henan, China
Source: Front Microbiol. 2026 Feb 4;17:1758643. doi: 10.3389/fmicb.2026.1758643 (PMC12913533; doi:10.3389/fmicb.2026.1758643)
Supplement: Supplementary file 1 [file Table_1.DOCX]

Supplementary Material

# Supplementary Data

Table S1. The serovars of Salmonella strains isolated from diarrhea patients, food, and environment in Henan, China from 2018 to 2022.

Table S2. Characterization of 68 S. Kentucky ST198 isolates and their antimicrobial resistance profiles.

Table S3. Antimicrobial resistance rates of 68 S. Kentucky ST198 isolates against 15 different antimicrobial agents.

Table S4. Antimicrobial resistance profiles and distribution of resistance genes among 68 S. Kentucky ST198 isolates. 1 indicates the presence of the resistance gene, while 0 indicates the absence.

Table S5. Basic information and QRDR mutation type on genomes of 168 S. Kentucky ST198 isolates downloaded from the NCBI database used for phylogenetic analysis.

Table S6. The difference in prevalence rates of resistance genes among Chinese clades ST198-1 and ST198-2.

Table S7. SNP distance matrices for 68 S. Kentucky ST198 isolates.
